# A Biomimic Reconstituted High-Density-Lipoprotein-Based Drug and p53 Gene Co-delivery System for Effective Antiangiogenesis Therapy of Bladder Cancer

**DOI:** 10.1186/s11671-015-0965-5

**Published:** 2015-07-08

**Authors:** Qiaohong Ouyang, Zhongxiang Duan, Guangli Jiao, Jixiao Lei

**Affiliations:** Department of Nuclear Medicine, The First Affiliated Hospital, Chinese PLA General Hospital, Beijing, China

**Keywords:** Bladder cancer, Reconstituted high-density lipoprotein, Candesartan, p53, Co-delivery, Antiangiogenesis therapy

## Abstract

A biomimic reconstituted high-density-lipoprotein-based drug and p53 gene co-delivery system (rHDL/CD-PEI/p53 complexes) was fabricated as a targeted co-delivery nanovector of drug and gene for potential bladder cancer therapy. Here, CD-PEI was utilized to effectively condense the p53 plasmid, to incorporate the plasmid into rHDL, and to act as an antitumor drug to suppress tumor angiogenesis. The rHDL/CD-PEI/p53 complexes exhibited desirable and homogenous particle size, neutral surface charge, and low cytotoxicity in vitro. The results of confocal laser scanning microscopy and flow cytometry confirmed that SR-BI-targeted function induced specific cytoplasmic delivery and high gene transfection efficiency in MBT-2 murine bladder cells. In addition, rHDL/CD-PEI/p53 complexes co-delivering CD and p53 gene achieved synergistic angiogenesis suppression by more effectively downregulating the expression of vascular endothelial growth factor (VEGF) messenger RNA (mRNA) and protein via different pathways in vitro. In vivo investigation on C3H/He mice bearing MBT-2 tumor xenografts revealed that rHDL/CD-PEI/p53 complexes possessed strong antitumor activity. These findings suggested that rHDL/CD-PEI/p53 complexes could be an ideal tumor-targeting system for simultaneous transfer of drug and gene, which might be a new promising strategy for effective bladder cancer therapy.

## Background

Bladder cancer is one of the most common cancers. There were approximately 70,980 cases diagnosed with bladder cancer in the USA in 2009, of which 14,330 patients would likely succumb to the disease [1]. Gene therapy has garnered significant attention as a therapeutic approach for bladder cancer. From a clinical point of view, this disease is an ideal target for gene therapy [2]. Efficient delivery of genetic material to the required cells within a patient without significant toxicity and side effect in gene therapy requires an ideal delivery vector, which has been extensively studied for several decades [3]. Reconstituted high-density lipoprotein (rHDL) is the synthetic form of the endogenous human HDL. Both rHDL and endogenous human HDL possess similar physicochemical properties. In the past decades, rHDL has been successfully developed as a scavenger receptor class B type 1 (SR-BI)-targeting gene carrier [4], which displayed promising application potential in vivo.

Antiangiogenic therapy has been well recognized as an effective antitumor strategy [5]. Among various angiogenesis-related growth factors, vascular endothelial growth factor (VEGF) has been demonstrated to be a major contributor to angiogenesis that occurs in many solid tumors including breast, bladder, and prostate cancers [6]. It has been reported that p53 gene therapy could inhibit tumor-associated angiogenesis by downregulating VEGF expression [7]. On the other hand, angiotensin II type 1 receptor (AT_1_R), a widely overexpressed receptor in various neoplastic cells, was recognized to play an important role in tumor angiogenesis and progression. There was increasing evidence that the AT_1_R blocker (ARB) candesartan (CD) exerted beneficial effects on tumor progression by competitively inhibiting the AT_1_R signaling pathway and downregulating VEGF expression [8].

Co-delivery systems simultaneously transporting anticancer drug and gene into the same cancer cells by multifunctional nanovectors may provide a new paradigm in cancer treatment. In this study, an rHDL-based system was developed for effective p53 gene delivery and combined antiangiogenesis therapy in a bladder cancer model. CD-PEI was first synthesized and then employed to construct a lipophilic core of rHDL (Lipos/CD-PEI). The cationic CD-PEI was served to condense the p53 plasmid to formulate Lipos/CD-PEI/p53 complexes. Finally, functional protein apoA-I was introduced to eventually assemble the delivery system (rHDL/CD-PEI/p53 complexes).

## Methods

### Cell Culture and Animal Model

The MBT-2 murine bladder cell line was cultured in DMEM (Gibco, USA) supplemented with 10 % FBS (HyClone, USA), 100 U/ml penicillin, and 100 μg/ml streptomycin in a humidified atmosphere of a 95 % air/5 % CO_2_ incubator at 37 °C. Male C3H/He mice (7 weeks old) were maintained at standard conditions with free access to food and water. All animal experiments were conducted in strict accordance with the National Institute of Health Guide for the Care and Use of Laboratory Animals. The MBT-2-tumor-bearing mice models were established by subcutaneous inoculation of the MBT-2 cell suspension to the flank of mice.

### Synthesis of CD-PEI

CD-PEI was prepared according to a previous report [9] and characterized by ^1^H NMR.

### Preparation of rHDL/CD-PEI/p53 Complexes

#### Preparation of Lipos/CD-PEI

A thin-film dispersion method was employed to construct Lipos/CD-PEI as previously reported [10] with some modifications. Briefly, 60 mg of PC, 6 mg of cholesterol, and 12 mg of CE were dissolved in 2 ml of organic solvent (chloroform:methanol = 1:1, *v*/*v*), and the solvent of lipid solutions was evaporated with a rotary evaporator at 30 °C until a thin film was formed. The trace solvent residue was finally removed with a stream of nitrogen gas. Five hundred microliters of CD-PEI solution (10 mg/ml), 50 μl of sodium cholate solution (30 mg/ml in phosphate-buffered saline (PBS) buffer), and Tris buffer (0.1 M KCl, 10 mM Tris, 1 mM EDTA, pH 8.0) were added to dissolve the thin film. The mixture was vortexed thoroughly for 5 min, followed by ultrasonication using an ultrahomogenizer (JY92II, Ningbo, China) until a clear suspension was obtained. The dispersion was then filtered through a 0.22-μm filter and dialyzed to remove the free sodium cholate (MWCO 7500 Da, 2 L × 3). Finally, the prepared Lipos/CD-PEI complexes were collected and stored at 4 °C until further use.

#### Preparation of Lipos/CD-PEI/p53 Complexes

The p53 plasmid was dissolved in PBS buffer to obtain a final concentration of 0.1 mg/ml and then added dropwise into the above-prepared Lipos/CD-PEI complexes with vortex to formulate Lipos/CD-PEI/p53 complexes.

#### Preparation of rHDL/CD-PEI/p53 Complexes

Lipos/CD-PEI/p53 complexes were incubated with 100 μl of apoA-I solution (30 mg/ml in PBS buffer) to form rHDL/CD-PEI/p53 complexes under 600-rpm stirring at 25 °C for 8 h. The prepared rHDL/CD-PEI/p53 complexes were obtained and stored at 4 °C until further use.

### Particle Size, Zeta Potential, and Morphology Observation

The particle size and zeta potential of Lipos/CD-PEI/p53 and rHDL/CD-PEI/p53 complexes were measured by a Malvern Zetasizer. The morphological observation was performed by transmission electron microscopy.

### Cytotoxicity, Cellular Distribution, and Transfection Studies

The MBT-2 cells were seeded in plates at a density of 1 × 10^5^ cells/ml and incubated until 70–80 % cell confluence before being subjected to experiments.

The cytotoxicity of the complexes was measured by MTT assay. PEI 1.8K/pDNA (*w*/*w* = 10, pDNA indicates the non-functional plasmid), Lipos/CD-PEI/pDNA, and rHDL/CD-PEI/pDNA complexes containing various concentrations of PEI were co-cultured with cells for 24 h.

The pDNA was labeled with the fluorescent dye YOYO-1 and employed to construct complexes as mentioned in “Preparation of rHDL/CD-PEI/p53 Complexes.” After incubation with the complexes at 37 °C for 2 h, cells were treated with 50 nM LysoTracker Red for 30 min and rinsed three times with PBS before staining with Hoechst 33342 (10 μg/ml). The cellular distribution of complexes was observed by confocal laser scanning microscopy.

Transfection of PEI 25K/pEGFP-C3 (*N*/*P* = 10), Lipos/CD-PEI/pEGFP-C3, and rHDL/CD-PEI/pEGFP-C3-complex-mediated reporter gene pEGFP-C3 in MBT-2 cells was qualitatively and quantitatively investigated as described previously [11]. The expression of green fluorescent protein (GFP) in cells was observed under an inverted fluorescence microscope, and the transfection efficiency of complexes was quantified by GFP fluorescence intensity and GFP-positive cells using flow cytometry.

### PCR, Western Blotting, and ELISA Assays

PCR, western blotting, and ELISA assays were conducted according to a previous report [12].

### In Vivo Antitumor Assay

In vivo antitumor efficacy of rHDL/CD-PEI/p53 complexes was evaluated on MBT-2 tumor xenograft models. All MBT-2-tumor-bearing nude mice were weighed and randomly divided into four groups (*n* = 6). All the formulations were administrated via tail vein at a dose of 30 μg CD and/or 50 μg p53 gene/mouse. The measurement of tumor volumes and the injection of formulations were repeated every 2 days for 2 weeks. At the end of treatment, all mice were sacrificed and their tumor tissues were harvested. The tumor tissues were pictured and then subjected to hematoxylin and eosin (H&E) staining.

## Results and Discussion

### Characterization of CD-PEI

The conjugation of CD with PEI was conducted via amidation reaction. The cationic amido groups of PEI were employed to condense the plasmid, and the highly hydrophobic CD was introduced to incorporate the PEI/pDNA complexes with the hydrophobic core of rHDL through hydrophobic interaction. Here, the CD-PEI served not only to package the plasmid but also to act as a linker to integrate the plasmid with rHDL. The chemical structure of CD-PEI was confirmed by ^1^H NMR in D_2_O. Compared with the spectrum of PEI, the proton peaks of –NHCH_2_CH_2_– from CD-PEI appeared at 2.2–3.3 ppm, whereas PEI only appeared at about 2.7 ppm. Moreover, the peaks at *δ* 1.2–1.5 ppm (t, –CH3) and *δ* 6.3–7.1 ppm (m, Ar-H) were assigned to CD. These results provided decisive evidences that CD was successfully grafted to the PEI chain.

### Particle Size, Zeta Potential, and Morphology Observation

**Fig. 1:**
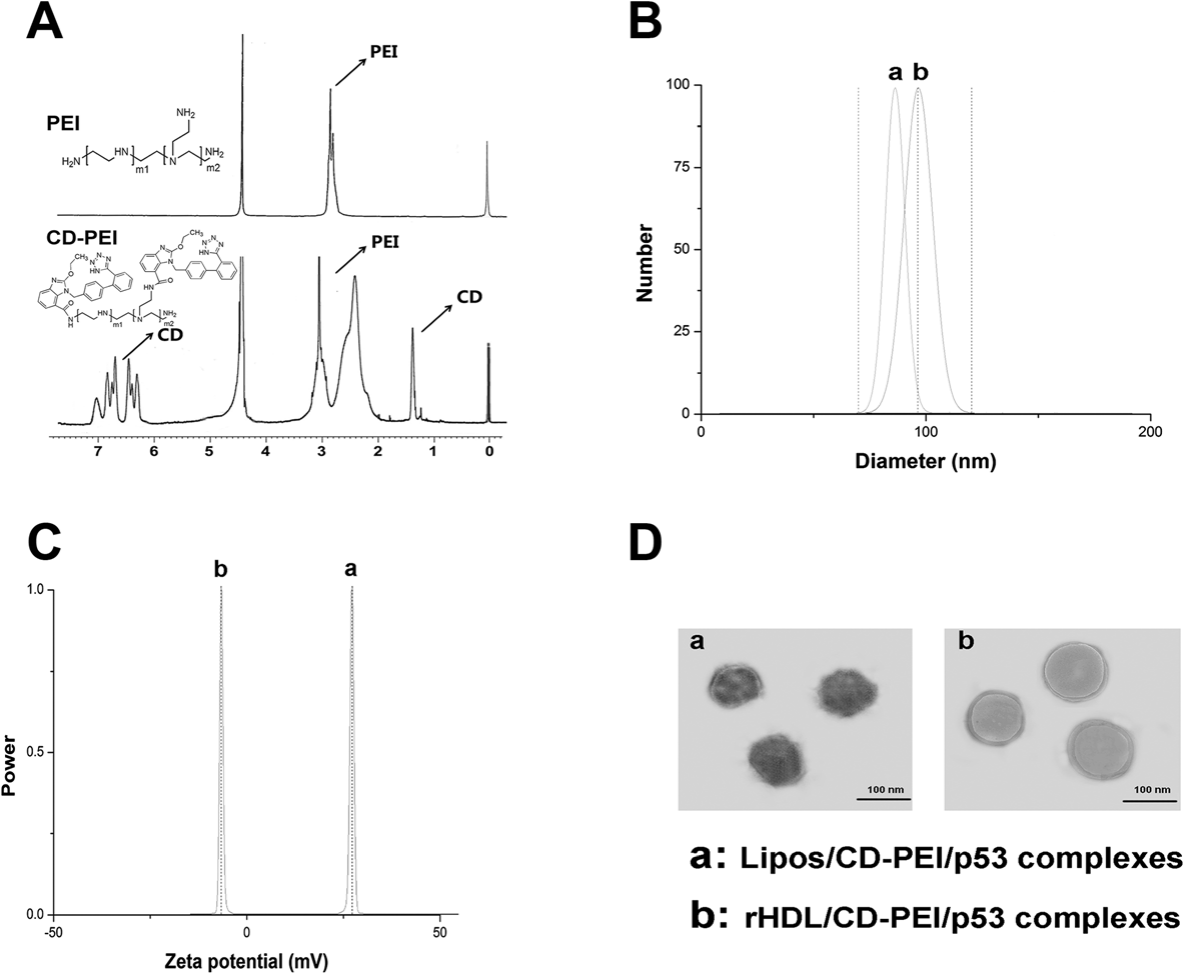
**a** ^1^H NMR of PEI and CD-PEI, **b** particle size, **c** zeta potential, and **d** TEM of Lipos/CD-PEI/p53 and rHDL/CD-PEI/p53 complexes. *Scale bars* 100 nm

The particle size and zeta potential are in great relation to the performance of the delivery system, which should be carefully tuned to achieve the optimal therapeutic effect in cancer therapy. Multiple researches have demonstrated that the biodistribution behavior and cellular uptake efficiency of complexes are relevant to their particle size and zeta potential [13, 14]. In general, a small size usually leads to preferable cellular uptake and superior therapeutic effect of particles, for they can be readily recognized and transported by the corresponding receptor or channel [12]. On the other hand, it is well established that the positively charged particles tend to interact with negatively charged proteins in the blood and extracellular matrix, which might lead to preferable uptake by the liver instead of targeting cells and could be an obstacle for the effective transfection of plasmid [15]. Herein, the particle size and zeta potential of Lipos/CD-PEI/p53 and rHDL/CD-PEI/p53 complexes were analyzed. As shown in Fig. 1b, both Lipos/CD-PEI/p53 and rHDL/CD-PEI/p53 complexes showed a nanoscale size under 100 nm. Comparing the particle size of these two complexes, a minor increase was observed in the rHDL/CD-PEI/p53 complexes group, which indicated the successful coating of apoA-I protein. This conclusion was also confirmed by the significant difference of zeta potential between them (Fig. 1c). Moreover, both Lipos/CD-PEI/p53 and rHDL/CD-PEI/p53 complexes displayed a spherical shape and compacted structure with good dispersity, as evidenced by the representative TEM image in Fig. 1d, which further proves the formation of compact nanosized particles.

### Cytotoxicity, Cellular Distribution, and Transfection Study

**Fig. 2:**
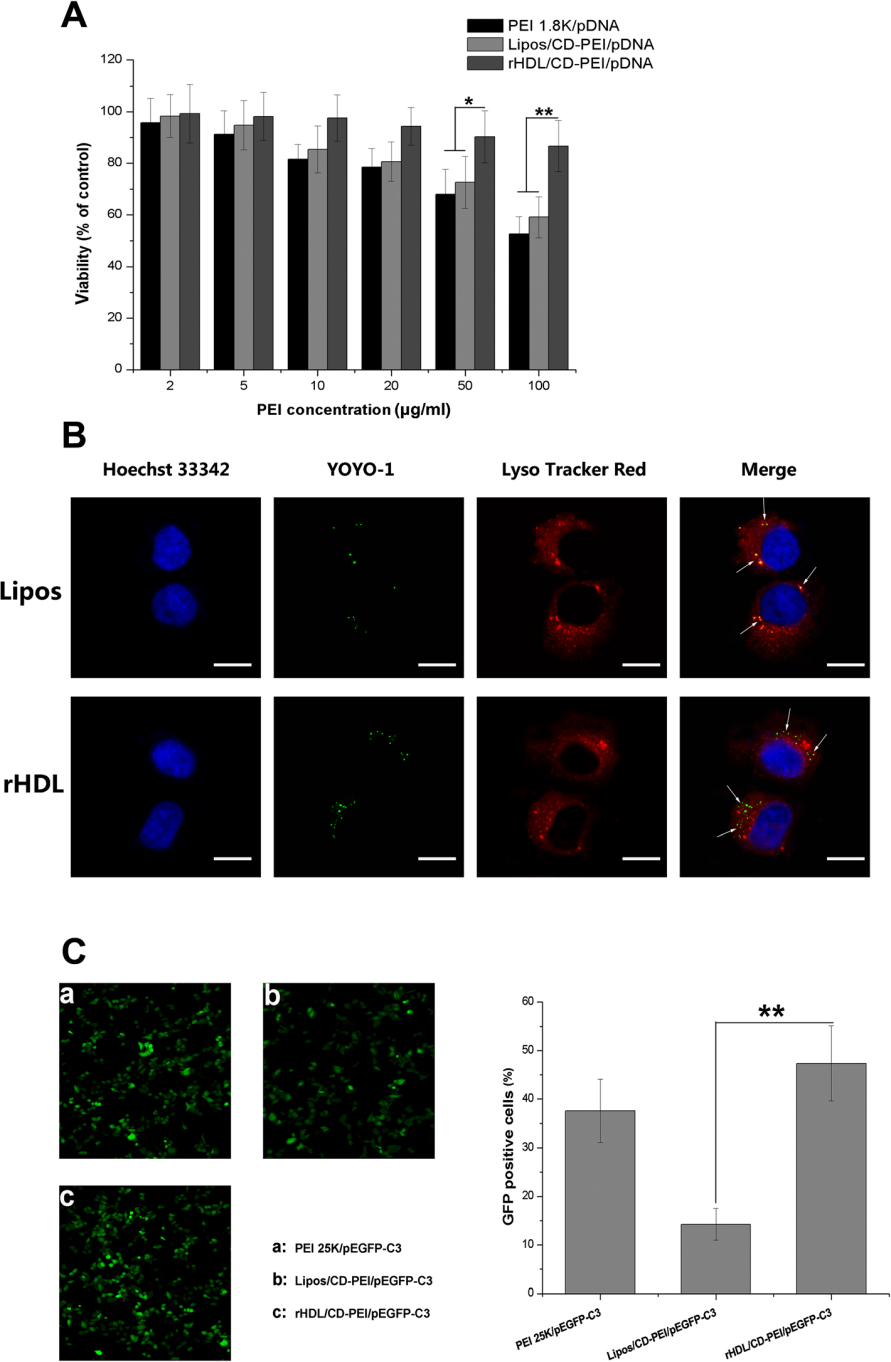
**a** Cell viabilities of MBT-2 cells incubated with different complexes at various PEI concentrations. **b** Intracellular trafficking of the YOYO-1-labeled rHDL/CD-PEI/pDNA complexes in MBT-2 cells by CLSM. **c** In vitro GFP expression mediated by PEI 25K/pEGFP-C3 (*N*/*P* = 10), Lipos/CD-PEI/pEGFP-C3, and rHDL/CD-PEI/pEGFP-C3 complexes in MBT-2 cells. Results are expressed as mean ± SD (*n* = 5). *P < 0.05 and **P < 0.01. *Scale bars* 20 μm

PEI is one of the most effective non-viral gene vectors owing to its condensed positive charges. However, the positive charges may interact with a negatively charged cell membrane and cause severe cytotoxicity both in vitro and in vivo [11]. The cytotoxicity of PEI 1.8K/pDNA (*w*/*w* = 10), Lipos/CD-PEI/pDNA, and rHDL/CD-PEI/pDNA complexes was evaluated against MBT-2 cells by MTT assay. Cells were treated with complexes containing various PEI concentrations ranging from 2 to 100 μg/ml. As presented in Fig. 2a, significant inhibitory effects of PEI 1.8K/pDNA and Lipos/CD-PEI/pDNA complexes were observed. PEI 1.8K/pDNA and Lipos/CD-PEI/pDNA complexes displayed 47.26 and 40.87 % mortality on MBT-2 cells at 100 μg/ml, respectively, which might be related to their considerable positive charges. In contrast, compared with PEI 1.8K/pDNA and Lipos/CD-PEI/pDNA complexes in all concentrations, the cell viability of rHDL/CD-PEI/pDNA complexes was higher. Especially at the PEI concentration of 100 μg/ml, the serious cytotoxicity of Lipos/CD-PEI/pDNA complexes was dramatically decreased after apoA-I protein shielding, indicating that rHDL as a biomimic delivery vector could lower the cytotoxicity of PEI. Moreover, the pDNA concentrations adopted in the following studies were all lower than 100 μg/ml; as a result, rHDL/CD-PEI/pDNA complexes could be identified as a safe delivery system for both in vitro and in vivo application.

It has been reported that rHDL could specifically deliver its encapsulated payload into the cytoplasm via the non-endocytic pathway [16]. By employing confocal laser scanning microscopy to localize the endosome and encapsulated plasmid, we hope to explore the cellular distribution of rHDL/CD-PEI/pDNA complexes and, in turn, explain their transfection mechanism. As depicted in Fig. 2b, the red fluorescence dots represented endosomes labeled with LysoTracker Red and the nuclei were stained with Hoechst 33342 (blue color). The intracellular green fluorescence dots induced from YOYO-1-labeled pDNA were utilized to indicate the location of the encapsulated payload of different complexes. After 2 h of incubation, significant differences were observed between the cellular distribution of Lipos/CD-PEI/pDNA and rHDL/CD-PEI/pDNA complexes. Most the green dots in the Lipos/CD-PEI/pDNA group merged with a red color (yellow), suggesting the payload of Lipos/CD-PEI/pDNA complexes was undergoing the endocytosis process. In contrast, none of the payloads in the rHDL/CD-PEI/pDNA group yielded a yellow color, indicating they all existed in the cytoplasm. We speculated that upon recognition of apoA-I by SR-BI, the cell membrane would undergo membrane reorganization, resulting in the formation of a non-aqueous “channel” and subsequent cross-membrane transfer of CD-PEI/pDNA cargo from rHDL to cytosolic compartments without internalization of the intact rHDL/CD-PEI/pDNA complexes. Bypassing the endocytosis process will guarantee the integrity of the CD-PEI/pDNA, which is beneficial to the transfection of the plasmid.

In order to determine the influence of non-endocytotic delivery effect on the transfection efficiency of rHDL/CD-PEI/pEGFP-C3 complexes, the in vitro transfection and expression of GFP were qualitatively and quantitatively analyzed by inverted fluorescence microscopy and flow cytometry, respectively. PEI 25K/pEGFP-C3 complexes with the *N*/*P* ratio of 10 were employed as a positive control. As displayed in Fig. 2c, compared with Lipos/CD-PEI/pEGFP-C3 complexes, the fluorescence image of rHDL/CD-PEI/pEGFP-C3 complexes showed a significant increase in transfection efficiency, which was even stronger than that in PEI 25K/pEGFP-C3 complexes. A similar conclusion could also be reached from the quantitative analysis using flow cytometry. The GFP-positive cells in rHDL/CD-PEI/pEGFP-C3 complexes were 3.24-fold higher than those in Lipos/CD-PEI/pEGFP-C3 complexes and were even 1.26-fold higher than those in PEI 25K/pEGFP-C3 complexes. The discrepancy of transfection capacity between Lipos/CD-PEI/pEGFP-C3 and rHDL/CD-PEI/pEGFP-C3 complexes demonstrated that the SR-BI-receptor-mediated non-endocytotic delivery of CD-PEI/pEGFP-C3 contributed to the evaluated transfection efficiency of rHDL/CD-PEI/pEGFP-C3 complexes.

### Synergistic Therapeutic Effect of CD and p53

VEGF is a potent and specific mitogen for endothelial cells, which has been considered as the most important cytokine in angiogenesis and has proved to be a suitable target for cancer therapy. Hence, the expression of VEGF messenger RNA (mRNA) and protein in MBT-2 cells transfected with rHDL/CD-PEI/p53 complexes was evaluated by quantitative reverse transcription PCR (qRT-PCR), western blotting, and ELISA assays, respectively.

**Fig. 3:**
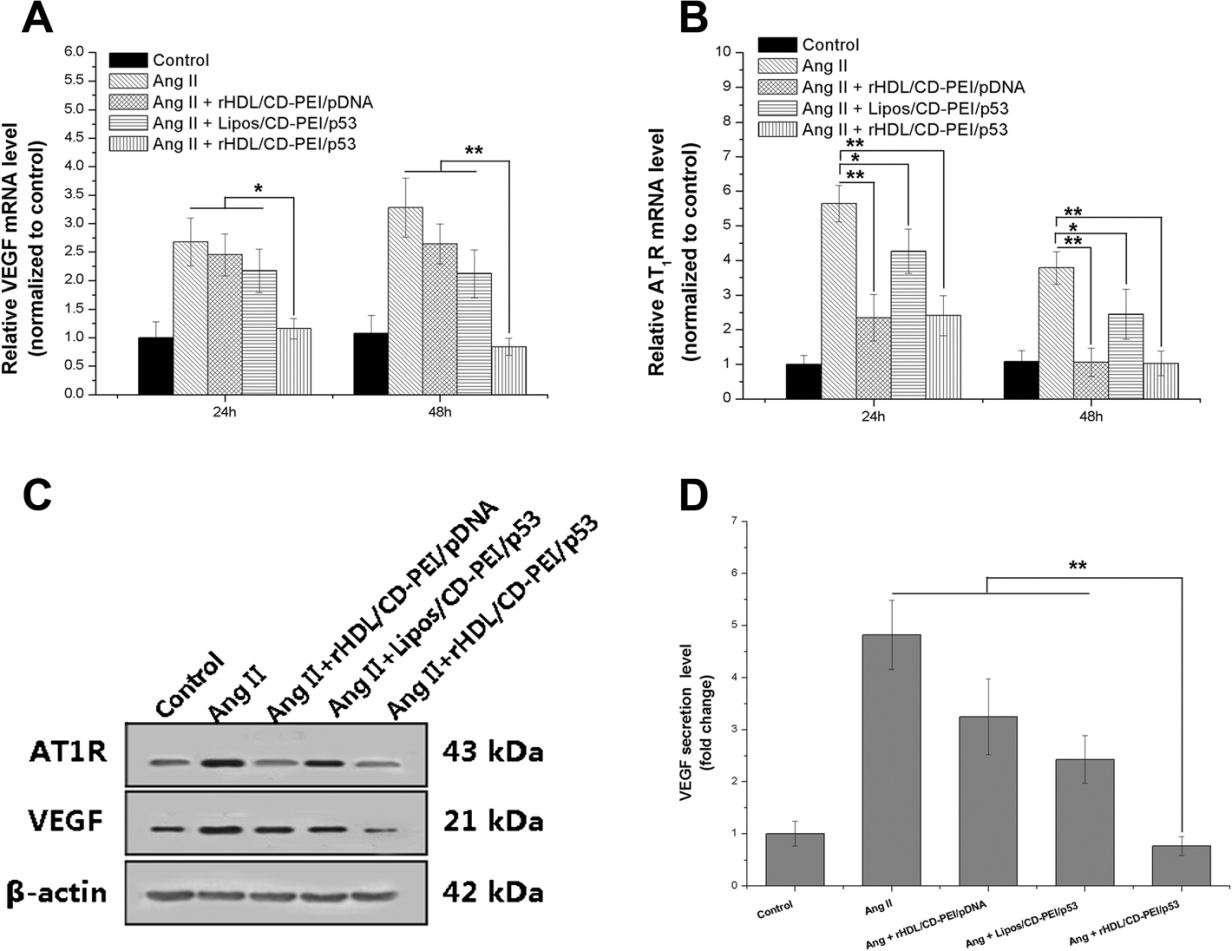
Synergistic therapeutic effect of rHDL/CD-PEI/p53 complexes in suppressing VEGF and AT_1_R expression in MBT-2 cells. Ang II (100 nM) was utilized to simulate an in vivo tumor microenvironment with high Ang II concentration. PCR assay of the suppression on VEGF (**a**) and AT_1_R (**b**) mRNA levels. **c** Western blotting assay. **d** ELISA assay. Results are expressed as mean ± SD (*n* = 5). *P < 0.05 and **P < 0.01

High Ang II concentration was employed to upregulate VEGF and AT_1_R expression [17]. As shown in Fig. 3a, treatment with Lipos/CD-PEI/p53 or rHDL/CD-PEI/pDNA complexes for 24 and 48 h respectively resulted in the lowered level of VEGF mRNA in MBT-2 cells. More importantly, rHDL/CD-PEI/p53 complexes which simultaneously delivered CD and p53 gene into the same cells further potentiated such suppression effect in VEGF mRNA level. It was noted that the VEGF mRNA level in cells treated with rHDL/CD-PEI/p53 complexes was 2.91-fold lower than that in Ang-II-stimulated cells, even lower than the basal level of VEGF mRNA in untreated cells after 48 h of treatment, regardless that both Ang II and endogenous mutant p53 upregulated VEGF expression [18, 19]. The synergy effect of the two therapeutic agents (CD and p53 gene) was further supported by the reduced VEGF protein expression observed by western blotting assay (Fig. 3c). On the other hand, ELISA (Fig. 3d) analysis showed that, at 48 h post-transfection, rHDL/CD-PEI/p53 complexes as a co-delivery system more dramatically downregulated the VEGF secretion from cells induced by Ang II and endogenous mutant p53 compared with an untargeting delivery system (Lipos/CD-PEI/p53) or a mono-delivery system (rHDL/CD-PEI/pDNA complexes), which was consistent with the abovementioned qRT-PCR and western blotting assays. All these results strongly demonstrated that effective co-delivery of CD and p53 gene into MBT-2 cells using rHDL/CD-PEI/p53 complexes achieved synergistic effect in suppressing overexpression of the angiogenesis-related gene VEGF, which might ultimately restrain tumor-associated angiogenesis.

Furthermore, Ang II reportedly upregulated VEGF expression through AT_1_R and inhibition of AT_1_R downregulated the Ang-II-dependent VEGF expression [20]. To confirm whether CD, an ARB, inhibited VEGF expression by downregulating AT_1_R expression, qRT-PCR and western blotting analysis were conducted to assess the expression level of AT_1_R mRNA and protein. Figure 3b, c shows that Lipos/CD-PEI/p53 complexes exhibited the highest expression of AT_1_R mRNA and protein, which might be related to their poor tumor-targeting ability. However, it was worth mentioning that rHDL/CD-PEI/pDNA complexes achieved almost the same level of AT_1_R mRNA and protein expression to rHDL/CD-PEI/p53 complexes, regardless of the incorporated plasmid. Taking the results from VEGF and AT_1_R expression analysis into consideration, it was evidenced that it was CD (not p53 gene) that specifically reversed Ang-II-inducible VEGF overexpression by downregulating AT_1_R expression.

Taken together, these results demonstrated that downregulation of VEGF expression was punctuated via different pathways (Ang II-AT_1_R and p53-dependent pathways) by which CD and p53 gene act synergistically.

### In Vivo Antitumor Efficacy and Tumor Angiogenesis Suppression

**Fig. 4:**
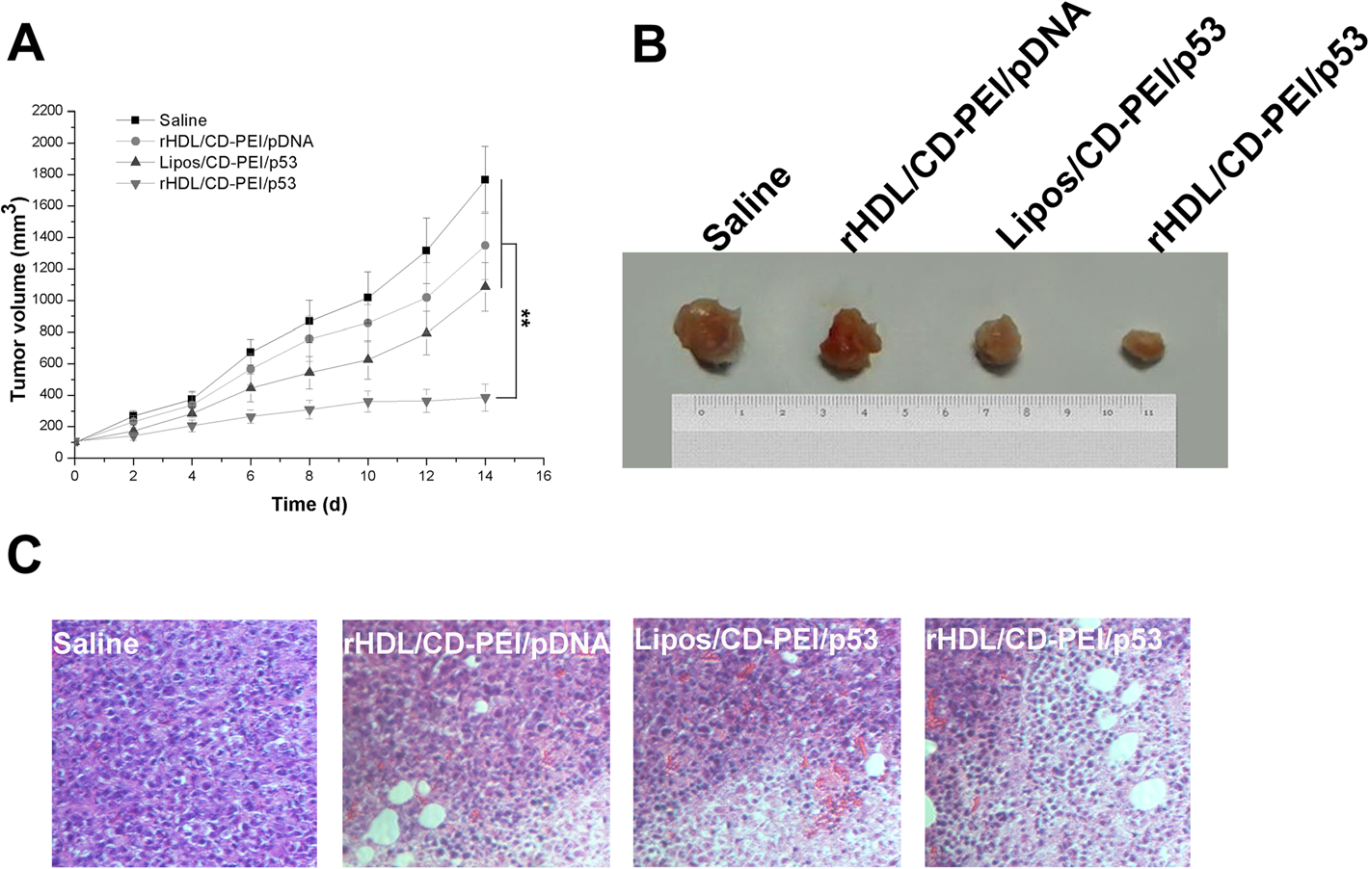
The tumor volume curve (**a**), typical images of ex vivo tumor tissues (**b**), and H&E staining (×200) of tumor tissues (**c**) of MBT-2-tumor-bearing C3H/He mice after intravenous administration of saline and different complexes, respectively. The measurement of tumor volumes and the injection of formulations were repeated every 2 days for 2 weeks. Results are represented as mean ± SD (*n* = 6). **P < 0.01

The potential in vivo synergistic antitumor efficacy of the rHDL/CD-PEI/p53 co-delivery system was further assessed in MBT-2-xenografted C3H/He mice. As shown in Fig. 4a, although tumor growth was suppressed to some extent after administration of the untargeting delivery system (Lipos/CD-PEI/p53) or the mono-delivery system (rHDL/CD-PEI/pDNA complexes), the combined therapy of CD and p53 gene appeared to be much more potent when the animals were treated with rHDL/CD-PEI/p53 complexes, for the final tumor volumes in the rHDL/CD-PEI/p53 group were merely 385.07 ± 86.19 mm^3^. This conclusion was also confirmed by the typical images of ex vivo tumor tissues (Fig. 4b).

The therapeutic efficiency was also detected by H&E staining of tumor tissues. The results were in accord with the tumor growth inhibition. As displayed in Fig. 4c, almost all tumor cells were seen arranging in nests and exhibiting typical tumor cell morphology in the saline group. In contrast, various degrees of coagulative necrosis were observed in different treatment groups. There was a clear boundary between the necrotic region and non-necrotic region. In the necrotic region, tumor cells were more heterogenous with high cellular polymorphism; severe damage was seen with nuclear pyknosis, cell necrosis, and cell lysis; and to some extent, cell fragments were also observed. Among all the treatment groups, the rHDL/CD-PEI/p53 group presented the most serious coagulative necrosis with the largest area of eosinophilic staining.

All the above results could be due to the synergetic anticancer mechanism of CD and p53 gene. Overall, the rHDL/CD-PEI/p53 complexes we constructed had great potential as co-delivery systems for efficient cancer therapy via synergistic mechanism.

## Conclusions

In this study, a biomimic reconstituted high-density-lipoprotein-based drug and p53 gene co-delivery system (rHDL/CD-PEI/p53 complexes) based on SR-BI targeting delivery and combined tumor therapy strategy was successfully constructed for simultaneously tumor-targeted delivery of CD and p53 gene. Combined CD and p53 gene therapy using a biomimic rHDL-based co-delivery system might be an effective strategy for improved antiangiogenic treatment in cancer, which deserves further investigations towards the thorough mechanism of action in vivo and potential clinical application.
